# Serum C-Reactive Protein (CRP), Target for Therapy or Trouble?

**Published:** 2007-02-07

**Authors:** Virginia B. Kraus, Joanne M. Jordan

**Affiliations:** 1 Duke University Medical Center, Durham, NC 27710; 2 Thurston Arthritis Research Center, University of North Carolina, Chapel Hill, NC 27599

**Keywords:** C-reactive protein, osteoarthritis, cardiovascular disease risk, inflammation

## Abstract

High sensitivity serum C-reactive protein (hs-CRP) has come into clinical use as a marker of risk for cardiovascular disease (CVD). In addition to a role as a marker of disease, CRP has also been implicated in the pathogenesis of CVD. Specific small-molecule inhibitors of CRP have recently been developed with the intent of mitigating cardiac damage during acute myocardial infarction. However, the use of CRP, both as a risk marker and a disease target are controversial for several reasons. Serum hs-CRP concentrations can be elevated on the basis of genetics, female gender, and non-Caucasian ethnicity. It is not clear, in these contexts, that elevations of hs-CRP have any pathological significance. As a non-specific indicator of inflammation, CRP is also not a specific indicator of a single disease state such as cardiovascular disease but elevated concentrations can be seen in association with other comorbidities including obesity and pulmonary disease. In sharp contrast to the proposed inhibition of CRP for cardiovascular disease treatment, the infusion of CRP has been shown to have profound therapeutic benefits for autoimmune disease and septic shock. The balance between the risks and benefits of these competing views of the role of CRP in disease and disease therapy is reminiscent of the ongoing controversy regarding the use of non-steroidal anti-inflammatory drugs (NSAIDs) for musculoskeletal disease and their cardiovascular side effects. Soon, NSAIDs may not be the only agents about which Rheumatologists and Cardiologists may spar.

## Commentary

C-reactive protein (CRP) is produced by the liver as part of the ‘reorchestration’ of hepatic gene expression in response to inflammation and infection (Black et al. 2004). An extremely sensitive acute phase reactant, CRP concentrations increase rapidly in serum and often exceed the reference range by 1000 times or more (Mortensen, 2001). Its rapid synthesis after infection suggests it contributes to host defense (Black et al. 2004). Barring recent infections, changes in disease state, or stress, CRP baseline concentrations are reported to be relatively steady with minimal diurnal or seasonal variation (Meier-Ewert et al. 2001). Moreover, unlike the cytokines, IL-6, IL-1β, and TNF-*α* that elicit CRP production from the liver (Mortensen, 2001), this protein has a relatively long plasma half-life (19 hours) and is quite stable in vitro (Aziz et al. 2003).

C-reactive protein was named for its ability to precipitate the “C” polysaccharide extracted from the pneumococcal cell wall (Black et al. 2004). Synthesis of the protein is now known to be stimulated in response to many pathogens including gram-positive (Mold et al. 1981) and gram-negative pathogens, fungi, and malarial parasites (Volanakis, 2001; Szalai, 2002). By binding to specific ligands of the pathogen’s cell wall, CRP activates the classical complement pathway and provides a means of defense against the invading pathogen. The finding of homologous CRP-like pentameric proteins, called pentraxins, in numerous vertebrates, as well as invertebrates (Magor and Magor, 2001), suggests it is an ancient element of an innate host immune defense strategy dependent upon the ability to opsonize pathogenic ligands (Tharia et al. 2002). Unlike the activation of complement by immunoglobulin, complement activation initiated by CRP is limited to C1–C4 by the complement-control protein, factor H (Giannakis et al. 2003; Du Clos, 2002; Giannakis et al. 2001). Therefore, CRP promotes phagocytosis of particles without generating a strong inflammatory response (Du Clos, 2002).

C-reactive protein also exhibits a distinct anti-inflammatory activity indicated by its protective effects against endotoxic shock, allergic encephalitis, inflammatory alveolitis, nephrotoxic nephritis, and systemic lupus erythematosus (SLE) (Black et al. 2004; Rodriguez et al. 2005; Szalai et al. 2000). This activity is believed to be mediated, at least in part, by the immunosuppressive cytokine IL-10, whose expression is induced by CRP’s binding to Fcγ receptors on macrophages (Ogden and Elkon, 2005). In addition, CRP appears to play a very important role in preventing autoimmunity (Du Clos and Mold, 2004; Szalai et al. 2002; Russell et al. 2004) by targeting apoptotic and necrotic cells for removal. Of note, many of the autoantibodies commonly associated with SLE are directed against the major CRP ligands reported in the literature, namely, chromatin, histones, fibronectin, small nRNPs, and laminin (Du Clos et al. 1991; Black et al. 2004); this suggests for at least this autoimmune disease, a failure of the normal CRP clearance mechanisms. However, debate continues over whether CRP is primarily a passive indicator of inflammatory events, a “culprit” mediating disease (Rattazzi et al. 2003; Bisoendial et al. 2005), or nature’s own immunosuppressant, functioning to limit tissue damage, modulating acute inflammation, and preventing autoimmunity.

The development of robust assays of superior sensitivity compared to those for basic CRP measurement, has allowed the identification of patients with low levels of inflammation. These high-sensitivity CRP (hs-CRP) assays have led to increasing use of this protein in the study of the inflammatory nature of many chronic diseases such as atherosclerosis (Ridker, 2004; Armani and Becker, 2005). With the recognition that inflammation plays a role in CVD and precedes myocardial infarction, numerous reports have emerged with plausible explanations for an association between hs-CRP and CVD (Pepys and Hirschfield, 2003) and for the characterization of hs-CRP as a robust and independent predictor of future cardiovascular events (Verma, 2004). The expanding interest in inflammation and its relation to CVD resulted in a 2002 workshop sponsored by the American Heart Association (AHA) and Centers for Disease control (CDC), and guidelines for the use of hs-CRP in the assessment of risk of such events have been proposed (Pearson et al. 2003). This consensus panel issued a statement regarding interpretive ranges for hs-CRP for assessment of risk for CVD (< 1 mg/L low risk group, 1–3 mg/L average risk group, and > 3 mg/L high risk group) (Pearson et al. 2003). Since then at least 945 clinical laboratories across the country have begun performing hs-CRP testing as an assay to assist in the assessment of CVD risk. However, the values on which these categories are based have been derived almost exclusively from Caucasian European and European American reference populations (Anand et al. 2004).

CRP is strongly associated with obesity, and weight loss has been shown to decrease CRP in nine of ten studies in which it has been evaluated (Dietrich and Jialal, 2005). Race and gender also strongly influence serum hs-CRP concentration (Khera et al. 2005). In Dallas County, characterized as a typical multiethnic U.S. urban population, the median hs-CRP level is 30% higher in blacks than in whites, and almost twice as high in women as men (Khera et al. 2005). A new study demonstrated that cardiorespiratory fitness level, hormone replacement therapy use, and high-density lipoprotein cholesterol accounted for the gender difference in hs-CRP (Huffman et al. 2006). It is also increasingly evident that genetic factors, including apoE genotype (Marz et al. 2004) and polymorphisms in the hs-CRP gene (Russell et al. 2004; Szalai et al. 2002; Suk et al. 2005), regulate basal hs-CRP concentrations. The substantial variability in hs-CRP concentrations in people of different ethnic origins, led Anand et al. to conclude that uniform hs-CRP cut-points were not appropriate for defining vascular risk across diverse populations (Anand et al. 2004). Our findings in an ethnically diverse population with comorbidities point to similar conclusions. In this cohort of 670 individuals (49% African American and 58% female), mean ln hs-CRP was higher in African-Americans and in women (p < 0.0001) and was strongly correlated with body mass index (r = 0.401, p < 0.0001), but not with age (r = 0.008, p = 0.841) (Jordan et al. 2002). In addition to ethnicity and body mass index, ln hs-CRP was also independently associated with chronic pulmonary disease. We further evaluated a subset of individuals in Johnston County, North Carolina, without self-reported cardiovascular disease and with a low ten-year risk for cardiovascular disease based upon the Framingham cardiovascular disease risk score (Wilson et al. 1998). Notwithstanding low risk by the Framingham score, the majority of these individuals were categorized as moderate or high risk for cardiovascular disease based upon serum hs-CRP concentration; women (even after excluding hormone replacement users) and individuals with osteoarthritis chiefly comprised this high-risk group based on hs-CRP (Kraus et al. 2006).

Controversy also exists regarding the potential for CRP to act as a mediator of atherothrombotic disease (Pepys and Hirschfield, 2003; Pepys, 2005). Although bacterial recombinant human CRP has been shown to induce a massive acute phase response in humans (Bisoendial et al. 2005), these results, and possibly most of the proinflammatory activities ascribed to CRP, may be attributable to contamination of CRP preparations with lipopolysaccharide, endotoxin, or the preservative, sodium azide (Pepys et al. 2005). Moreover, the description of CRP as a pathogenic agent underlying cardiovascular disease (Pearson et al. 2003; Armani and Becker, 2005) contrasts sharply with the generally protective and anti-inflammatory action of CRP (Volanakis, 2001) described above. The large population-based Dallas Heart study has recently reported that hs-CRP was not independently associated with atherosclerotic burden defined by either coronary artery calcification on cardiac electron-beam computed tomography scans or by abdominal aortic plaque on magnetic resonance images (Khera et al. 2006). Other recent assessments, with careful control for traditional risk factors for CVD, suggest that minimal improvement in CVD risk prediction is provided by hs-CRP over conventional risk factors (Sepulveda and Mehta, 2005). The relative risk ratio (RR) of hs-CRP for CVD is estimated to be much lower (RR=1.45) than originally reported (Levinson et al. 2004); used alone, hs-CRP has been estimated to have a very low positive predictive value (≤0.86%) for predicting CVD (Levinson et al. 2004; Levinson, 2005).

A small-molecule inhibitor of CRP, perhaps the first of many to come, has recently been designed and synthesized (Pepys et al. 2006). This inhibitor abrogated the increase in infarct size and cardiac dysfunction produced by injection of human CRP in a rat model of acute myocardial infarction. Thus, the rationale for targeting CRP is based upon the ability of human CRP to increase myocardial and cerebral infarct size in rats subjected to coronary or cerebral artery ligation, respectively. However, this may be nothing more than an epiphenomenon related to the inability of the rat isoform of the native complement-control protein, factor H, to interact with human CRP. Of note, rat CRP does not activate rat complement, nor cause these deleterious effects in the rat myocardial infarction model. In the experiments described above, human CRP was required to activate rat complement.

Ethnicity and gender exert strong influences on serum hs-CRP concentration and confound the prediction of CVD risk based upon hs-CRP. Caution is advised in the use of serum hs-CRP for predicting CVD or its inhibition for treating CVD until the consequences of CRP inhibition are better understood, and further conclusive evidence is available demonstrating a pathologic role for CRP in CVD, taking into account the biology of native interacting factors, and evaluating the pathologic significance of CRP elevations due to ethnicity, gender and genetics.
